# A Proof of Concept of a Mobile Health Application to Support Professionals in a Portuguese Nursing Home

**DOI:** 10.3390/s19183951

**Published:** 2019-09-12

**Authors:** Márcia Esteves, Marisa Esteves, António Abelha, José Machado

**Affiliations:** Algoritmi Research Center, University of Minho, Campus Gualtar, 4470 Braga, Portugalabelha@di.uminho.pt (A.A.); jmac@di.uminho.pt (J.M.)

**Keywords:** business intelligence, elders, health information and communication technology, health professionals, mobile health, nursing homes, smart health

## Abstract

Over the past few years, the rapidly aging population has been posing several challenges to healthcare systems worldwide. Consequently, in Portugal, nursing homes have been getting a higher demand, and health professionals working in these facilities are overloaded with work. Moreover, the lack of health information and communication technology (HICT) and the use of unsophisticated methods, such as paper, in nursing homes to clinically manage residents lead to more errors and are time-consuming. Thus, this article proposes a proof of concept of a mobile health (mHealth) application developed for the health professionals working in a Portuguese nursing home to support them at the point-of-care, namely to manage and have access to information and to help them schedule, perform, and digitally record their tasks. Additionally, clinical and performance business intelligence (BI) indicators to assist the decision-making process are also defined. Thereby, this solution aims to introduce technological improvements into the facility to improve healthcare delivery and, by taking advantage of the benefits provided by these improvements, lessen some of the workload experienced by health professionals, reduce time-waste and errors, and, ultimately, enhance elders’ quality of life and improve the quality of the services provided.

## 1. Introduction

Over the past few years, the world has been witnessing a huge demographic change: the population is aging at an alarming rate. In fact, the statistics regarding the aging population are concerning since, compared to the growth of the whole population, it is estimated the elderly population is growing twice as quickly [1]. Consequently, this problem has been a matter of concern for many countries since it is posing several challenges to healthcare systems worldwide [1,2,3]. Thus, as a consequence of the rapidly aging population, the costs of elderly care and the number of elders in nursing homes have been increasing [1,3].

The harsh reality is that many countries are experiencing a growth in the proportion of elders and, consequently, an increase of the number of service requirements for them that, at the moment, they are not able to meet. Thus, due to the high demand for more and better medical services for the elderly, there is a need to evaluate the state of these services and assess the need for improvements.

Portugal is not an exception to this concern. In fact, Portugal is, at the moment, one of the countries with the largest aging population in the world [4] and, similar to other countries, this situation has been negatively affecting several aspects of elderly care. In this sense, one of the major challenges resulting from this situation is the increasing number of elders in nursing homes. Over the past few years, nursing homes vacancies have been filling up at a quick rate, making the search for a place in one of these facilities a massive challenge for many elders and families in Portugal [5,6].

Additionally, health professionals working in nursing homes are, more than not, overloaded with work since they are often few compared to the high number of elderly people [7,8]. In addition to the aging population, one of the main factors causing this situation is the lack of investment and resources in these facilities. In this context, nursing homes generally use unsophisticated and rudimentary methods, namely paper, to record information and to clinically manage residents [9,10]. Naturally, the paper-management of data is more error-prone and time-consuming since the risk of misplacing or losing information is much higher. Moreover, health professionals constantly need to return to the nursing stations to retrieve and record information, leading to a higher risk of forgetting information or writing information in the wrong place.

Thus, the current need to fill the lack of resources and access to technology in nursing homes to solve some of the problems faced by them and ultimately improve the nursing care delivered is apparent. In fact, nursing homes could greatly benefit from the introduction of technological advancements, such as HICT. The use of HICT, which refers to any form of electronic solution that allows manipulating, managing, exchanging, retrieving, and storing digital information in healthcare settings, has dramatically and positively changed the medical practice and is certainly here to stay [11,12,13].

Technologies encompassed in HICT have rapidly become a natural and indispensable part of healthcare settings due to their many advantages, namely to enhance the management, access, and sharing of information; to improve the quality, safety, and efficiency of healthcare delivery and its outcomes; to reduce the occurrence of errors and adverse events; to support the decision-making process; to decrease time-waste; and to improve productivity in healthcare systems [9,10,11,12,13,14,15]. In fact, the use of HICT in medical contexts enables turning traditional healthcare towards smart healthcare, which consists in the use of technology to improve healthcare delivery and the quality of services. Nevertheless, despite the well-known benefits of HICTs, nursing homes have been lagging behind in adopting them due to the lack of investment and effort by these facilities to adapt to technological improvements [9,10,11,15,16].

Thereby, considering all the above mentioned, this manuscript aims to describe and evaluate a proof of concept of a mHealth application developed for health professionals, more specifically the doctors and nurses working in a Portuguese nursing home. The solution was developed to introduce technological improvements in the facility and to support the health professionals in their daily tasks and at the point-of-care, namely to manipulate and have access to information as well as to schedule, perform, and record their job-related tasks. Moreover, clinical and performance BI indicators were also defined to help health professionals to make more informed and evidence-based decisions.

It is important to mention that a mobile solution was chosen since a single hand-held device, which can be used anywhere and at any time, can allow accessing and manipulating information at the point-of-care. In this sense, the novelty of this project resides in the need to solve some of the challenges faced by a nursing home suffering from the consequences of the aging population and the absence of HICT. Additionally, the lack of literature and an integrated body of knowledge on the use of HICT in nursing homes shows that there is still much work that needs to be done in this area.

Regarding the structure of this document, Section 2 corresponds to the state of the art in which the body of knowledge related to this project is described. Then, in Section 3, the research methodologies that were selected to successfully conduct this project are approached. Afterwards, in Section 4, the developments tools that were chosen to develop the mobile application, namely the database, web services, and interfaces, and to create examples of the BI indicators are identified as well as their advantages. Section 5 gives a brief description of the Portuguese nursing home, i.e., of the case study, for which the solution was developed in order to have a better understanding of the main challenges faced by the institution. Then, the results achieved regarding the database, web services, interfaces, and BI indicators developed are presented in Section 6. A brief discussion of the results obtained is presented in Section 7. Finally, in Section 8, the main conclusions and contributions achieved are identified and future work is presented.

## 2. State of the Art

In this section, the general background related to the research area of this project is presented in order to offer a deeper understanding about the novelty and relevance of this project, namely about how mHealth and BI can positively impact and be beneficial for healthcare facilities, more specifically, for the nursing home used as a case study in this study. Furthermore, the ethical issues associated with the use of HICT in healthcare contexts are described since they were taken into account during all stages of the development of this project. Finally, works related to the project carried out in this study are also addressed.

### 2.1. The Impact of Mobile Health in the Healthcare Industry

In recent years, the rapid expansion of mobile technology, i.e., of technology that can be used “on-the-move”, has been affecting several industries, and the healthcare industry is not an exception [17,18,19]. In fact, the ubiquitous presence of mobile devices, such as smartphones and tablets, and the rise in their adoption have led to the growth in the number of mobile applications. In this sense, there is currently a wide range of mobile applications that offer a variety of features and, more recently, mobile health applications have been expanding due to their potential to improve healthcare delivery [18,19,20].

In this context, the use of mHealth, i.e., mobile devices and applications to support the medical practice, has been transforming several aspects of the healthcare industry and proving to be quite promising and beneficial for health professionals, namely to help them execute their daily tasks, to manage and monitor patients, to access and manage clinical data, and to enhance the decision-making process, among others [17,19,21,22,23]. However, mHealth has not only been advantageous for healthcare providers but also for the consumers, allowing them to strengthen their communication with healthcare organizations [20,24]. Therefore, the main benefits of mHealth are as follows [17,20,21,24]:

- Convenient and faster accessibility to information since all data are gathered in a single source, which can be used “on-the-move”;
- Reduction of time-waste since health professionals can manipulate information at the point-of-care, not having to interrupt their workflow and go to another location to do so;
- Faster and better decision-making process, since health professionals can have access to up-to-date information at the point-of-care, leading to more informed and based decisions;
- Faster and improved communication since mHealth helps connect all the professionals distributed across the healthcare organization;
- Help healthcare organizations to strengthen their communication with healthcare consumers by providing information to them at any given moment through appointment reminders, test result notifications, diagnostics, and disease control, among others;
- Decrease errors and adverse events; and
- Improve quality of healthcare delivery and services.

It is important to mention that the use of mobile applications in healthcare settings is not intended to replace desktop applications, which can be more powerful and less restrictive than mobile applications, but to complement them and, especially, to enhance outcomes at the point-of-care [17]. In fact, in situations where rapid information exchange is needed, where information should be entered at the point-of-care, and where health professionals are constantly on the move and have, therefore, less time to spend on computers, mobile technology is highly beneficial compared to desktop applications [21]. For instance, health professionals working in nursing homes could greatly benefit from mobile technology since they are constantly in motion and have little time to spend on computers, which are often located in nursing stations far away from the residents.

Therefore, the undeniable benefits of mHealth show that a higher investment should be done in its adoption as it can improve the quality of healthcare delivery. However, mHealth applications should only be developed after truly understanding the needs of the intended users in order to develop high quality and accurate applications and avoid their underutilization [17,19,21,22].

### 2.2. Business Intelligence Transforms Clinical Information into Valuable Information

Business intelligence corresponds to a set of methodologies, applications, processes, technologies, and analytical tools that enables to gather, store, manipulate, process, and analyze data in order to gain new and relevant information used by organizations to make informed and evidence-based decisions [13,25,26,27]. In the healthcare industry, BI tools are essential to analyze the clinical data constantly generated in order to obtain new knowledge used as evidence to support the decision-making process [25,26,27,28]. Thereby, BI has emerged as a solution to make use of the complex and huge amounts of information gathered daily in organizations, offering analytical tools able to turn these data into meaningful, useful, and valuable information and, thus, make faster, informed, and evidence-based decisions [27,29,30,31].

Furthermore, through the knowledge obtained, organizations are able to gain a deeper understanding and insight on their performance and highlight problem areas and opportunities, enabling them to plan and perform improvements if necessary [25,26,28,30,32]. Regarding the healthcare industry, applying BI technology to electronic health records (EHRs) helps improve healthcare delivery and its outcomes; reduce the occurrence of errors, adverse events, and costs; and give economic value to the large amounts of clinical data generated daily, which otherwise would be a burden to healthcare organizations [25,26,28,30,32,33].

**Figure 1:**
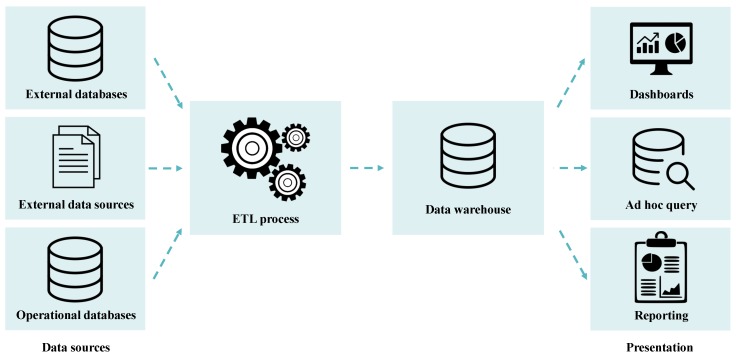
General architecture of the business intelligence process (adapted from [34]).

The general architecture of the business intelligence process is illustrated in Figure 1.

As shown in Figure 1, the components encompassed in the BI process include the following [13,34,35,36]:

- Extract, transform, and load (ETL) process: enables extracting data from multiple sources, clean and normalize these heterogeneous data to make them consistent and unambiguous, and load the transformed data into a data warehouse (DW);
- Data warehousing process: enables building adequate DWs able to structure data and facilitate their analysis; and
- Visualization, analysis, and interpretation of the data loaded into the DW: enables obtaining new knowledge previously unknown to an organization. Thus, for this purpose, various analytical tools and applications can be used, namely data mining tools and applications able to create charts, reports, spreadsheets, and dashboards, among others.

Despite the opportunities and positive effects BI brings to organizations, this technology has not yet attained its full potential and maturity in the healthcare industry [13,30]. However, the benefits of BI tools in healthcare settings are indisputable and have, thus, continuously been explored through the years.

### 2.3. Ethical Issues in Medicine

Without any doubt, the use of HICT, mHealth, and BI in the healthcare industry has been greatly beneficial and advantageous for healthcare organizations since these technologies have the potential to enhance the quality of the care delivered. However, despite the many benefits and opportunities offered by these technologies, they are not without flaws. In fact, challenges may arise from the implementation and use of solutions based on them, more specifically, ethical issues.

Nowadays, healthcare organizations produce daily vast amounts of EHRs and other types of data related to both the patients and the organization. However, since these data are stored in health information systems, patients are fearful that their confidentiality and privacy are compromised and not guaranteed, since, compared to the traditional paper-based management of data, technological advancements have made accessing data and violating privacy easier [22,37,38]. Additionally, the EHRs of the patients can be consulted by various health professionals across the organization, which can be problematic for patients who do not want their sensitive information shared and viewed by other professionals [37,39].

In this sense, privacy issues and patient confidentiality should always be taken into account and safeguarded while developing technological solutions. In fact, if the privacy and confidentiality of the users are not protected and ensured, some of them may not want to use HICT solutions [39]. Furthermore, legal issues may arise if sensitive information of the users is disclosed without their consent and if their privacy is lost. Therefore, it is important to define data access policies in order to only give information access to authorized users [38,39]. Nonetheless, implementing security protections remains a difficult task to perform, but it should always be taken into account and viewed as a priority when developing HICT solutions [38].

On the other hand, regarding the introduction of mHealth solutions in healthcare settings, some health professionals remain hesitant regarding their use despite the many advantages and benefits provided by them. The main cause of this situation is the fact that many mHealth applications are currently being used without having a complete understanding of their effectiveness, accuracy, quality, and associated risks, which can, in extreme cases, impair healthcare delivery [17,22]. In this sense, best-practice standards should be followed to ensure the quality, accuracy, and safety of mHealth solutions during their design, development, and implementation [17,22,40]. Additionally, these applications should go through a rigorous set of validation and evaluation methods to guarantee their quality, accuracy, and safety in healthcare settings [17,22,40].

### 2.4. Related Work

Undeniably, the introduction of mobile devices and applications has been positively transforming several aspects of the medical practice and providing many benefits to healthcare facilities, namely the improvement of the quality of their services and healthcare delivery. Therefore, to shed light on the potential of mHealth, some existing works are presented in this subsection.

Nowadays, the vast majority of mHealth applications focus on specific health dimensions and are, thus, frequently oriented towards patients [41]. In this sense, there is currently an extensive amount of mHealth applications available in the market and they are being used to monitor patients both at home and in health facilities, to educate patients, to strengthen the communication between patients and health facilities, and to offer better access to health services, diagnosis, and treatment, among others [42].

In this context, it is possible to highlight several examples such as the mHealth monitoring system named iCare [43], which uses smartphones and wireless sensors to monitor elderly people in the comfort of their homes. This system is of particular interest since it enables remotely monitoring the elderly anywhere and at any time, providing different services according to the health conditions of each individual. Moreover, this system also acts as an assistant offering reminder, alarms, and medical guidance to the elderly. On the other hand, home-based telerehabilitation for people with multiple sclerosis was also addressed by Thirumalai et al. [44] through the development of a therapeutic exercise application named TEAMS, which provides different exercises and programs according to the multiple sclerosis level of the individual.

In the work of Parmanto et al. [45], a mHealth system called iMHere, which enables individuals with chronic conditions to perform preventive self-care tasks at home and to remotely communicate with clinicians without having to go to health facilities, is proposed. Finally, Bastos et al. [46] developed the SmartWalk project, which promotes healthy aging by enabling elderly people to have a more active lifestyle while being remotely monitored by health professionals. This project involved the development of a mobile application connected to sensors that collect data while the elderly user walks on a predefined route provided by the application. The health professionals are then able to analyze these data to suggest modifications to the route and, thus, improve the health of the elderly user.

However, despite the predominance of patient-centered mHealth solutions in the market, applications are also available for the management of health facilities and healthcare information and to assist health professionals. In this context, Doukas, Pliakas, and Maglogiannis [47] proposed a mobile healthcare information management system named @HealthCloud that enables medical experts as well as patients to manage healthcare information. Thus, by using this system, users are able to retrieve, upload, and modify medical content, such as health records and medical images. Moreover, the authors affirmed that the system enables managing healthcare data in a pervasive and ubiquitous way, leading to the reduction of medical errors since medical experts can effectively communicate between each other and have access to patient-related information during decision-making. Similarly, Landman et al. [48] developed a mobile application called CliniCam that enables clinicians to securely capture clinical images, annotate them, and finally store them in the EHR. Thus, this application enables making the images available to all credentialed clinicians across the hospital in a secure way. To this end, various security features were adopted, such as user authentication, data encryption, and secure wireless transmission.

Despite the existence of a large amount of patient-centered mHealth applications, the implementation of mobile technology for the management of health facilities, namely of nursing homes, and to assist health professionals and medical experts in their daily tasks remains to be properly addressed, whereby further research is needed. In this context, this project was performed as an answer for the lack of mobile solutions in nursing homes that focus primarily on the assistance of health professionals in their job-related tasks and management of the facility. Thus, due to the lack of applications similar to the one described in this manuscript, the health professionals working in the nursing home used as a case study were constantly consulted in order to develop a solution that answers to their needs. Furthermore, information gathered from the literature, namely from Landman et al. [48], was also essential to promote security features.

## 3. Research Methodologies

This project was sustained by a set of well-defined steps with the intention of ensuring its success and having an organized path to follow. In this context, the design science research (DSR) methodology was used since it is suitable for HICT research projects. Additionally, this methodology was used since the developed solution meets the needs of the health professionals working in the nursing home and is able to solve the problems faced by them. In fact, by introducing the solution into the nursing home, it is possible to substitute the paper-based management of information, support the decision-making process, reduce time-waste and the occurrence of errors and adverse events, and, consequently, lessen the work overload experienced by health professionals as well as improve the nursing care delivered.

The main purpose of the DSR methodology is to create and evaluate objects known as artifacts, or more specifically, solutions, developed in order to solve and address organizational problems [49,50,51]. In other words, the DSR methodology corresponds to a rigorous science research method that encompasses a set of techniques, principles, and procedures followed to design and develop successful solutions capable of solving problems faced by an organization and useful and effective to face the problems at hand [49,50,51].

**Figure 2:**
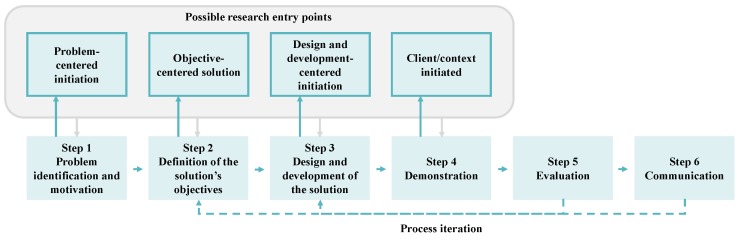
Schematic representation of the steps encompassed in the DSR methodology (adapted from [50]).

In this sense, the DSR methodology can be divided into six distinct steps, as illustrated in Figure 2.

Therefore, since the DSR methodology was used for the development of this project, the problems and challenges faced by the health professionals working in the nursing home used as a case study had to be identified in order to motivate the development of the solution. Thus, focus groups, semi-structured interviews, and questionnaires were made with the professionals working for the nursing home as well as for the hospital that manages the facility in order to gather valuable information capable of identifying and understanding the main challenges encountered by the health professionals. It is important to mention that the focus groups, semi-structured interviews, and questionnaires were performed with a group of ten participants, including nurses working in the nursing home as well as information and communication technology (ICT) professionals and other professionals working for both the nursing home and the hospital that manages the facility. The participants were selected based on their availability and since they were the most suitable to provide information concerning the challenges faced by the nursing home and the use of HICT in the facility. Furthermore, an observation of the case study was also performed to have a better understanding of the conditions of the nursing home.

Consequently, the objectives of the solution were defined according to the problems identified and, afterwards, the features and architecture of the solution were designed and developed. Once the solution was developed, it had to be demonstrated and evaluated through the execution of a proof of concept, which included a strengths, weaknesses, opportunities, and threats (SWOT) analysis and the technological acceptance model 3 (TAM3), in order to assess its usefulness, feasibility, and potential and if improvements and changes were needed. Additionally, this study also involved the communication of the problem and the solution to an audience, namely through the presentation of the solution to the health professionals and the writing of scientific papers.

A proof of concept was performed in order to carry out a thorough evaluation of the solution and to demonstrate its usefulness and potential. Therefore, a proof of concept enables to demonstrate in practice the concepts, methodologies, and technologies encompassed in the development of a solution. Additionally, it allows validating the developed solution towards the target audience and ensures that the solution provides all of the requirements initially proposed. On the other hand, besides being able to assess the usefulness, potential, and benefits of a solution, a proof of concept is also capable of identifying potential issues and threats associated with the solution.

Thus, the demonstration of the potential and feasibility of the mobile application involved the execution of a SWOT analysis to identify its strengths, weaknesses, opportunities, and threats. To this end, the TAM3 model was used to elaborate a questionnaire, which was performed with the health professionals working in the nursing home and the results obtained were used as a basis in the SWOT analysis. Thus, in this research project, the TAM3 model was followed to elaborate and design a questionnaire, which was performed with the users of the solution to assess their acceptance towards it.

Briefly, the TAM3 corresponds to a tool capable of predicting the acceptance of an information technology (IT) solution by users in an organization as well as the likelihood of this technology being adopted by them. To this end, the model considers that the acceptance and use of technology are affected by the internal beliefs, attitudes, and intentions of users and that their satisfaction towards IT results from the combination of the feelings and attitudes regarding a set of factors linked to the adoption of the technology [52,53,54]. Therefore, the attitudes and acceptance of users towards an IT solution influence and affect its successful implementation and use in an organization [55]. Thus, analyzing the acceptance of users towards a new IT solution is quite essential since the more accepting they are, the more willing they are to make changes and spend their time and effort to use the solution [55]. Organizations can then use the factors that affect the opinion of users towards the acceptance of a new IT solution and manipulate these factors to promote its successful use.

## 4. Development Tools

In this section, the development tools and technologies used to develop the solution are described as well as the reasons behind their selection and main advantages.

### 4.1. MySQL Relational Database Management System

Naturally, the development of any mobile application should include the definition and creation of a database, if one does not already exist, to store and manipulate data. In this sense, the database designed and developed for this project was created with MySQL.

MySQL is a relational database management system (RDBMS), meaning that it uses the relational model, in which several tables are logically related to each other through relations existing between them, as its database model [28,56]. Additionally, since it is a database management system (DBMS), MySQL enables defining, modifying, and creating a database as well as inserting, updating, deleting, and retrieving data from the database [56]. In addition, a DBMS offers controlled access to the database, namely a security system that blocks unauthorized users when they try to access the database, an integrity system that allows maintaining the consistency of data, a concurrency control system that allows shared access of data, a recovery control system that resets the database to its previous state in the case of a failure, and a catalog accessed by users to consult the descriptions of the data stored in the database [56].

For the development of this project, MySQL was chosen to define and create the database since it is a RDBMS as well as an open-source, fast, secure, reliable, and easy to use DBMS [28,57]. Additionally, the server in which the database had to be deployed and implemented, which belongs to the hospital that manages the nursing home, was already configured for this type of database, thus making MySQL the most appropriate choice.

### 4.2. PHP RESTful Web Services

The communication and interaction between the mobile application and the MySQL database was possible through the creation of RESTful web services, which were created using PHP. RESTful web services are based on the representational state transfer (REST) architecture, which is a client–server-based architecture, and depends on the hypertext transfer protocol (HTTP) protocol to convey the messages [58,59]. Thus, the REST architecture offers a set of principles on how data should be transferred over a network. RESTful web services are identified by uniform resource identifiers, which enable the interaction and exchange of messages with the web services over a network [58,59]. Moreover, by taking advantage of the specific features of HTTP, RESTful web services are able to GET, PUT, DELETE, and POST data

Thus, the web services were created to enable the mobile application to send requests to the database (via queries) and to send back to the application responses in the JavaScript object notation (JSON) format. The web services created enable to select data from the database as well as update and insert data. Consequently, to allow the communication between the mobile application and the web services, an Apache server was used, which is a HTTP server capable of receiving and sending HTTP messages.

PHP was chosen to develop the web services since it is an open-source, fast, and easy to use language. On the other hand, the server in which the web services had to be implemented was already configured for this programming language since other applications were developed for the hospital that manages the nursing home using PHP. Thus, taking into account the reasons mentioned above and to avoid maintenance and integration issues in the future, PHP revealed to be the most appropriate choice.

### 4.3. React Native JavaScript Framework

The interfaces of the mobile application were created using React Native, which is a JavaScript framework developed by Facebook for building native mobile applications, i.e., applications built for specific mobile platforms [60,61,62].

React Native was released in 2015 and is based on React, which is a JavaScript library used to build user interfaces and targets the web. However, React Native targets mobile platforms and enables developers to simultaneously develop and maintain one application that can be deployed to both iOS and Android [60,61]. Thus, developers do not need to develop distinct applications in order to target these two platforms. It is important to note that, although the mobile application built in this study was developed only for Android devices, choosing a cross-platform framework was still essential to allow its quick and easy development for iOS devices in the future.

In recent years, React Native has been proving to have a lot of potential as a cross-platform framework enabling developers to build native applications while having a high performance. On the other hand, React Native provides many other benefits, such as [63]:

- It is an open-source and free platform, making the development of mobile applications a lot easier since all documentation is available for free and it is community driven;
- Existence of a huge variety of third-party plugins and libraries to help and facilitate mobile development;
- Existence of a hot reload feature allowing developers to see updates without recompiling their application and updating its state;
- Existence of a live reload feature allowing developers to instantly reload their application without recompiling it;
- Straightforward and easy to use since it has a modular and intuitive architecture; and
- Has a great performance in mobile devices since it makes use of the graphic processing unit.

Thereby, all of the reasons mentioned above made React Native the most indicated choice to develop the interfaces of the mobile application. Furthermore, at the time of the development of this project other applications were being developed for the hospital that manages the nursing home using React and React Native. Thus, React Native revealed to be the obvious choice to avoid maintenance and integration issues in the future.

### 4.4. Power BI Business Analytics Platform

One of the objectives of this project was to identify and define clinical and performance indicators in order to make the decision-making process more evidence-based and accurate. However, it is important to mention that these indicators have not been created since the database does not have real data yet. Furthermore, in the future, it is envisioned to introduce them in a web application. Thus, to this end, Power BI was used to create examples of the clinical and performance indicators defined with fictitious data.

Power BI is a business analytics platform released in 2013 by Microsoft Corporation that provides BI tools to the users able to collect, analyze, visualize, and share data [64]. Thus, by aggregating data from various data sources, such as Excel, MySQL databases, and CSV files, among others, Power BI is capable of creating charts, reports, and graphs to obtain visuals and a better insight on the data [64].

On the other hand, Power BI is available in a desktop application, which is only executable on Windows, and in a cloud service [64]. Whereas the desktop application is used to model data and create reports, graphs, and charts, the cloud service is used to share and visualize them as well as create them. Therefore, when users need to perform data modeling, the desktop application is the best choice. However, to share dashboards, users need to use the cloud service.

Thus, the Power BI desktop application was used to create visual examples of the clinical and performance indicators defined. The choice of using this BI platform was due to the fact that it is a free, easy to use, and intuitive tool that enables to quickly create charts and graphs without too much effort and to visualize them in a simple and explicit way.

## 5. Case Study: A Portuguese Nursing Home

As already stated, this study consisted in designing and developing a mobile application for health professionals working in a Portuguese nursing home in order to assist them at the point-of-care, e.g., to schedule, perform, and record tasks and to have access, record, consult, and manipulate information, and to help them clinically manage the residents. It is important to mention that the nursing home used as a case study for this project is managed by a Portuguese hospital. Therefore, the professionals working for both the nursing home and the hospital were consulted throughout this project.

To have a better understanding of the relevance and motivation of this project, it was essential to identify the main issues and challenges faced by the health professionals and the nursing home. Therefore, focus groups, semi-structured interviews, and questionnaires were performed with the professionals working for both the nursing home and the hospital in order to obtain valuable information that could enlighten the main challenges faced by the nursing home. On the other hand, the case study was also subjected to observation so as to have a better understanding of its conditions.

Thus, the following challenges were identified:

- HICT or any other form of technological progress is not used in the nursing home. Although there is a computer in the nursing station, it is not used to record clinical information of the residents or even to schedule tasks. Therefore, there are no EHRs and health professionals use handwritten charts and medical records. Thus, since the information is stored on paper, the management of information is a lot more time-consuming, especially at the point-the-care, as the professionals have to consistently go back to the nursing station to manipulate information. Additionally, this situation can lead to a higher risk of losing, misplacing, or forgetting, information as well as documenting information in the wrong place.
- The job-related tasks of the health professionals are scheduled and documented in handwritten charts or boards. This situation is particularly problematic since it is more error-prone, confusing, and less organized.
- The nursing home does not have access to a wireless Internet connection. The health professionals can only have access to an Internet connection in the nursing station where the computer is located. This situation is especially challenging since it complicates the implementation of any kind of mHealth solution.
- The number of health professionals compared to the high number of elderly people is low. Consequently, at times, the health professionals are overloaded with work.
- There was a failed attempt to implement a web application. The web application aimed to shift from the paper-based to the computer-based management of data, allowing the health professionals to schedule tasks, document them, and record clinical information. However, the application was abandoned as it was time-consuming and not user-friendly.

In addition to the above mentioned, this project was also motivated by the fact that the health professionals revealed their need for a solution that would allow them to perform their daily tasks anywhere in the nursing home and in a more organized and faster way. Consequently, the need to design and develop a solution that could assist the health professionals at the point-of-care by allowing them to manipulate information anywhere in the facility was obvious. In this sense, a proof of concept of a mobile application designed and developed to enhance the care delivered and elders’ quality of life, reduce the occurrence of errors and time-waste, and ease some of the workload experienced by them was conducted.

## 6. Results

As mentioned above, the interfaces of the mobile application were developed using React Native, which is a JavaScript framework that enables building native mobile applications. It is important to state that, although React Native allows using the same code to deploy to both iOS and Android devices, the mobile application was only deployed for Android since Android devices are more affordable and common and are, therefore, more likely to be provided by the nursing home when the application is used in the future. However, if needed and after small modifications, the application can be quickly and easily deployed to iOS devices.

**Figure 3:**
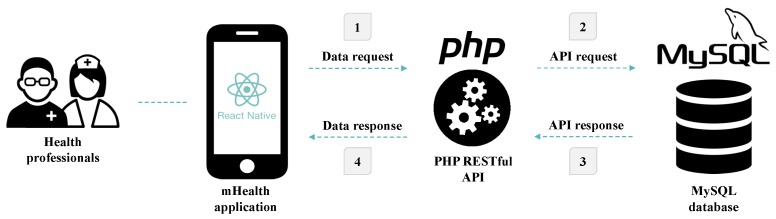
Schematic illustration of the architecture of the mobile application.

On the other hand, the MySQL RDBMS was also used to define and create the database. In this sense, SQL was the language used to manipulate and access the data stored in the database. Furthermore, to enable the communication and transfer of data between the mobile application and the database, RESTful web services were created using PHP. Therefore, the solution is divided into three distinct elements, each with a different purpose. Figure 3 illustrates the architecture and different interactions existing between the various elements of the mobile application.

At this point in time, the mobile application is fully developed, and the web services and the database are deployed in the server of the hospital that manages the nursing home. However, the solution is still being evaluated and tested by the health professionals. Moreover, the mobile application is not being used since the requirements, such as mobile devices and a reliable wireless Internet connection, have not yet been provided to the nursing home. Nevertheless, until the requirements are available to the nursing home, it is envisioned to continue improving the solution through the opinions and knowledge continuously provided by the professionals.

Finally, it must be mentioned that, during all stages of the design and development of this project, ethical issues were taken into account and safeguarded to guarantee that confidentiality issues do not arise as well as the quality, accuracy, and safety of the solution. In this sense, the health professionals were constantly consulted throughout the design and development of the solution in order to develop an accurate and high quality mobile application. Furthermore, data privacy and confidentiality were promoted with the implementation of a login through which only authorized users, namely the nurses and doctors, with encrypted login credentials, can have access to the information contained in the solution. On the other hand, the solution will only be accessed by being connected to an Intranet connection, i.e., the private network of the institution.

### 6.1. Database and RESTful Web Services Definition and Implementation

As mentioned above, the nursing home uses handwritten medical records and resorts to paper to manipulate information. Consequently, the facility did not have any database implemented prior to the development of this project. Therefore, before designing the interfaces of the mHealth application, a database had to be defined in order to allow the application to have access and store data. Thus, a MySQL relational database was defined and created taking into account the data that needed to be stored. Then, the database was deployed and implemented in the server of the hospital that manages the nursing home. However, it must be mentioned that the database remains to be populated with data related to the residents and the health professionals.

In this sense, a database composed of 49 tables was designed and created, allowing the storage of:

- Data related to the users of the mobile application: personal information of the health professionals (their full name, email, profile picture, telephone and mobile phone numbers, date of birth, institution identification number, and gender, among others) is stored as well as their login credentials.
- Personal data related to the residents (their full name, institution process number, bed and bedroom numbers, admission date, date of birth, profile picture, telephone and mobile phone numbers, and national health service number, among others) is stored.
- Personal data related to the informal caregivers and personal contacts of the residents (their full name, telephone and mobile phone numbers, relationship with the resident, and observations, among others) is stored.
- Clinical notes written by the doctors: The content of the note, the institution identification number of the professional who wrote the note, the resident’s institution process number, and the date and time of the creation of the note are stored.
- Nursing notes written by the nurses: Similar to the clinical notes of the doctors, the content of the note, the institution identification number of the professional who created the note, the resident’s institution process number, and the date and time of the creation of the note are stored.
- Clinical information related to the residents, namely their general evaluation (e.g., alcohol and tobacco consumption), usual medication, clinical history (e.g., existence of diabetes, diseases, allergies, and past surgeries and fractures), physical assessment (e.g., weight, height, blood pressure, heart rate, skin integrity, turgidity, and color, vision, and hearing), nutritional and eating patterns (e.g., type of diet, dentition, and use of a nasogastric tube), bowel and bladder elimination patterns (e.g., use of adult diapers or of a urinary catheter), physical activity patterns (e.g., strength of the limbs), sleeping patterns (e.g., insomnia problems and number of hours of sleep during the day and night), and general assessment made by the health professionals (e.g., emotional state or autonomy level) is stored.
- Data related to the wounds of the residents, namely the type of wound, pictures of the wound, and its location, treatments, and start and finish dates are stored. The evolution of the wounds is also documented through photos and observations provided by the health professionals. Additionally, the various treatments used throughout the evolution of the wound are stored.
- Periodic evaluations recorded by the health professionals (blood pressure, weight, heart rate, and axillary temperature) are stored. In this context, the date and time of the evaluation, the institution identification number of the professional who made the evaluation, and the resident’s institution process number are stored.
- Periodic evaluations of the capillary blood glucose of residents with diabetes are stored. Again, the date and time of the evaluation, the institution identification number of the professional who made the evaluation, and the resident’s institution process number are stored.
- The history of the medical and inpatient reports of the residents: The date, type, and a brief description of the report, among others, are stored.
- The nursing interventions scheduled by the health professionals through the identification of the type of nursing intervention, the scheduled and realization dates of the intervention, the resident’s institution process number, the institution identification numbers of the professionals who scheduled and performed the nursing intervention, and the state of the intervention, i.e., if the intervention was performed or not, are stored.
- Data related to the nursing home, namely the name of the institution and the bedroom and bed numbers existing in the nursing home, are stored.
- Technical data on the types and sizes of urinary catheters and nasogastric tubes available and types of wounds, injectable medications, nursing interventions, wounds location, and medical and inpatient reports, among others, are stored.

Afterwards, RESTful web services written in PHP with SQL queries were developed to allow the sharing of data between the frontend (the mobile application) and the backend (the database). In this sense, numerous web services were created to allow users to manipulate data from the database, namely to insert, update, and select data. Finally, similar to the database, the web services were deployed in the server of the hospital.

### 6.2. Mobile Application Features

After designing and developing the database and the web services, the interfaces and the features of the mobile application had to be designed and developed. For this purpose, React Native was chosen, as stated above.

At first, when the user, i.e., the health professional, launches the mobile application, he needs to sign up for an account if he does not have one. In this context, the user is requested to provide his login credentials and personal data. In this context, the user is requested to specify if he is a nurse or a doctor since these two user types have access to different features once signed in to the application. Then, once the user has provided his login credentials and his personal data, the data are stored into the database.

Alternatively, if the user already has an account, he can directly sign in to the mobile application with his login credentials. Finally, if his login credentials match with the ones stored in the database, the user is successfully signed in to the application, having access to the following features:

- Daily tasks: the user can consult the nursing interventions/tasks planned for the day and confirm or cancel their execution. Furthermore, the user is also able to consult the tasks that were already executed or cancelled. This feature is only available for nurses since, through interviews performed with the health professionals, it was concluded that doctors do not schedule tasks when present in the nursing home.
- Scheduled tasks: The user is able to consult the pending tasks, the cancelled tasks, and the finished tasks scheduled in the future, i.e., after the current date. Additionally, he can also cancel or confirm the execution of a task. For the same reasons mentioned above, this feature is only available for nurses.
- Plan of the nursing home: Both user types can consult the list of bedrooms existing in the nursing home. Then, by choosing one of the bedrooms, the user has access to the following information: the number of beds available and the name of the residents living in the bedroom. For each resident, the bed number is specified as well as the number of pending tasks associated with the resident for the day.
- Management of the residents: If the user is a nurse, he is able to manage the residents living in the nursing home. He can also view and edit their personal data as well as add new residents or disable a given resident if needed. Additionally, the user can view and edit the informal caregivers and personal contacts of each resident as well as add and remove contacts. However, if the user is a doctor, he is only able to view the personal data of the residents and the informal caregivers of each resident. Thus, doctors cannot insert new residents and informal caregivers, disable them, and edit their personal data.
- Clinical notes: If the user is a doctor, he is able to create new clinical notes and consult the clinical notes’ history of each resident. However, nurses are only able to view the clinical notes’ history of each resident since clinical notes can only be written by doctors.
- Nursing notes: If the user is a nurse, he is able to create new nursing notes and consult the nursing notes’ history of each resident. However, doctors are only able to consult the nursing notes’ history of each resident since nursing notes can only be written by nurses.
- Management of the clinical information of the residents: If the user is a nurse, he can manage, i.e., edit and view, the clinical information of the residents. However, doctors can only view the clinical information of the residents.
- Management of wounds: If the user is a nurse, he can manage the wounds of the residents and consult the wound history of each resident. More specifically, the user can insert new wounds for each resident as well as consult and record their evolution through photos and observations. Additionally, it is also possible to consult the history of the treatments used throughout the evolution of a wound and modify the current treatment if needed. Moreover, the user can also download a PDF file of the evolution of a given wound. However, doctors can only consult the wounds’ history of each resident, the evolution of each wound and of the treatments used, and download the PDF file of the evolution of the wound.
- Periodic evaluations: This feature is available to both users and allows them to add new periodic evaluations and consult the periodic evaluations’ history of each resident.
- Periodic evaluations of the capillary blood glucose: This feature is available to both users, enabling them to add new periodic evaluations of the capillary blood glucose for residents with diabetes. It is also possible to consult the history of the periodic evaluations of the capillary blood glucose of each resident with diabetes.
- Inpatient reports: This feature is available to both users and allows them to add new inpatient reports and consult the inpatient reports’ history of each resident.
- Medical reports: This feature is available to both users, allowing them to add new medical reports and consult the medical reports’ history of each resident.
- Planning of nursing interventions: This feature is only available for nurses, enabling them to schedule nursing interventions for each resident.
- Profile: This feature is available to both users, allowing them to have access and edit their personal data.
- Sign out: This feature is available to both users and allows them to sign out of their accounts.

### 6.3. Clinical and Performance Business Intelligence Indicators

To analyze and gain a deeper understanding of the overall performance of the nursing home and its health professionals as well as to improve the nursing care delivered and its outcomes, clinical and performance indicators were defined. However, at the moment, these indicators have not yet been created since the database does not have real data. Moreover, to create meaningful and valuable indicators, data should be gathered over a relatively long period of time, which is not the case at the moment. Furthermore, to have a better visualization and control over the indicators, it is envisioned to implement them in a web application and not in the mobile solution.

Thereby, in the future, when enough data are gathered, it is envisioned to create, at least, the following clinical and performance indicators:

- Percentage of nursing interventions realized per nurse: Pie chart indicator of the percentage of nursing interventions realized per nurse over a time horizon, for instance, per month and year. Thus, this indicator would enable highlighting if the nursing interventions are performed proportionately among the nurses working in the nursing home and if a certain health professional has a higher workload compared to others. Consequently, with the information obtained through this indicator, improvements and measures could be realized to have a better distribution of the nursing interventions between the nurses.
- Total of realized and unrealized nursing interventions per month: Stacked column chart indicator of the total of realized and unrealized (neither realized nor cancelled) nursing interventions. This indicator would help identify abnormalities in the number of unrealized nursing interventions as well as the months in which more tasks are performed or unrealized. Consequently, regarding the former, if too many nursing interventions are unrealized, it may suggest that the nurses are not performing their job as well as they should. For instance, it may shed light on wheather the nurses are overloaded with work, not having enough time to perform all of their tasks. On the other hand, regarding the latter, if some specific months are busier than others, more nurses could be present for each shift in order for the nursing interventions to be realized as scheduled.
- Variation of the capillary blood glucose of a given resident over time: Line chart indicator of the variation of the capillary blood glucose of a given resident over time. Thus, the health professionals would be able to have a better visualization of variation of the capillary blood glucose and, thus, more rapidly detect abnormalities and act on them. Additionally, this indicator could also be extended to other types of evaluations, namely to analyze the variation of the weight, blood pressure, heart rate, oxygen saturation, and axillary temperature of a given resident over time.
- Percentage of wounds per resident: Bar chart indicator of the percentage of wounds per resident over a time horizon, for instance, per month or year. Consequently, with this clinical indicator, the health professionals would be able to identify the residents with an abnormal amount of wounds and, thus, supervise them more closely so as to avoid and reduce the occurrence of wounds for these residents.
- Percentage of wounds per wound type: Donut chart indicator of the percentage of wounds per wound type over a time horizon, for instance, per month or year. Thus, through this clinical indicator, the health professionals would be able to identify if certain wound types occur more frequently than others. Consequently, according to the results obtained, further research and improvements could be realized so as to identify and reduce wound-causing factors.
- Percentage of nursing interventions realized annually per type of nursing intervention: Bar chart indicator of the percentage of nursing interventions realized annually per type of nursing intervention. Therefore, through this indicator, the health professionals would be able to identify and be aware of the nursing interventions that are not realized with the expected frequency. Hence, with this knowledge, the health professionals could perform these nursing interventions more frequently.

**Figure 4:**
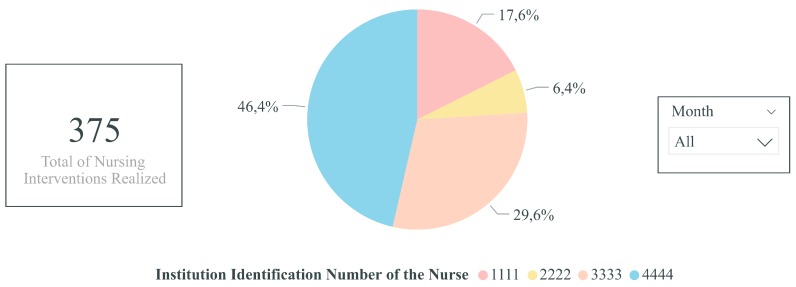
Indicator of the percentage of nursing interventions realized per nurse (created with fictitious data).

**Figure 5:**
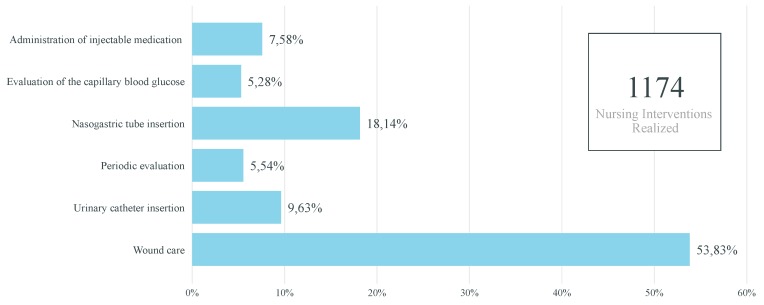
Indicator of the percentage of nursing interventions realized annually per type of nursing intervention (created with fictitious data).

**Figure 6:**
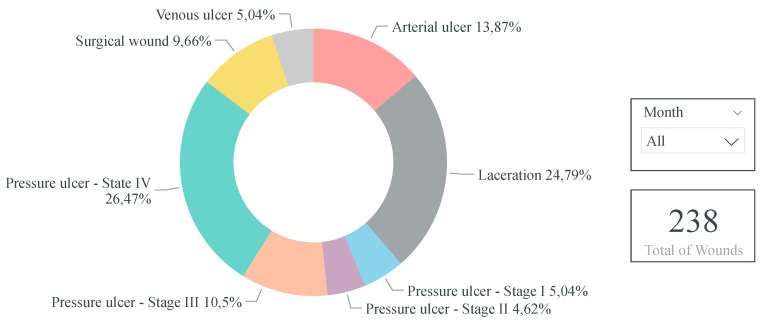
Indicator of the percentage of wounds per wound type (created with fictitious data).

Figure 4, Figure 5 and Figure 6 illustrate examples of some of the indicators mentioned above. Power BI was used with fictitious data.

## 7. Discussion

After the development of the mobile application, a proof of concept was performed to validate the usability, feasibility, and usefulness of the solution towards the target audience and to ensure that the solution provides all of the requirements initially proposed. Therefore, a SWOT analysis was elaborated to identify the strengths, weaknesses, opportunities, and threats related to the solution. To this end, a questionnaire based on the TAM3 was conducted with the health professionals working in the nursing home in order to assess their acceptability, i.e., how they accept and receive the mobile application, and its results were used as a basis in the SWOT analysis. Furthermore, this analysis was also based on personal opinion as well as valuable information obtained through semi-structured interviews and focus groups realized with the professionals working for both the nursing home and hospital.

It must be mentioned that the survey questionnaire was conducted with few health professionals. Thus, not enough results were obtained to be presented. However, in the future, it is intended to evaluate the mobile application with more health professionals and, thus, have a more complete evaluation.

The SWOT analysis performed is presented hereafter. The following strengths were identified:

- Decrease of time-waste and, consequently, an increase in productivity since the health professionals can have access and record information at the point-of-care, i.e., they do not need to constantly return to the nursing station;
- Decrease of the occurrence of errors since the solution reduces the risk of misplacing, losing, or forgetting information;
- Enhancement of the nursing care delivered and elders’ quality of life due to the decrease of errors and time-waste;
- Easier access and manipulation of information;
- Timely sharing and centralization of information;
- Optimization of the various processes occurring in the nursing home;
- Answer to the needs of the health professionals;
- Scheduling of tasks less confusing and more organized compared to hand-written boards;
- Reduction of the amount of paper generated daily with hand-written charts due to the shift from the paper-based to the computer-based management of data;
- Evidence-based and more accurate decision-making process since the health professionals can have access to information at the point-of-care;
- High usability since the mobile application has a simple, user-friendly, and intuitive design with well-defined paths and organized information;
- High adaptability since the solution can easily be implemented in other nursing homes; and
- High scalability since new features can easily be added and the mobile application can easily be maintained.

The following weaknesses can be pointed out:

- Need of a wireless Internet connection, which is not currently available in the nursing home;
- Need of mobile devices, namely mobile phones and tablets, in order to use the solution;
- Need to populate the database with real data, namely information of the residents and health professionals, which will require time resources;
- Need to train the health professionals before using the solution; and
- Need to wait a relatively long period of time before creating the clinical and performance indicators.

The opportunities of the solution are as follows:

- Introduction and implementation of the mobile application in other nursing homes;
- Enhancement of other processes due to the technological improvement of the nursing home; and
- Creation of clinical and performance indicators due to the elimination of the paper-based management of data and the storage of information in a database.

Finally, the following threats can be highlighted:

- Issues may emerge if a reliable wireless Internet connectivity is not available; and
- New systems and competition may arise due to the novelty of the solution, which approaches recent problems.

In light of the above mentioned, it is possible to affirm how beneficial and influential mHealth and BI are in healthcare organizations, namely to enhance the various processes occurring in them and, consequently, to improve the care delivered and patients’ quality of life. In fact, through the use of mobile applications, such as the solution described in this manuscript, the medical practice can be completely transformed as they allow rapid and convenient access to and manipulation of information at the point-of-care. Thus, for professionals constantly on the move, which is the case with the health professionals working in the nursing home used as a case study, a mHealth solution such as the one developed allows reducing time-waste since they do not need to interrupt their workflow, decreasing the occurrence of errors since the likelihood of forgetting or misplacing information is lower, and making faster and better decisions since they can have access to up-to-date information at the point-of-care making informed decisions. Furthermore, through BI tools, it is possible to use and analyze the huge amounts of data gathered daily in organizations in order to turn these data into valuable knowledge. In fact, the clinical and performance indicators defined in this research project enable highlighting problem areas and opportunities existing in the nursing home and shed light on the overall performance of the facility and its professionals.

Finally, regarding the ethical issues associated with the implementation of HICT in healthcare contexts, they were safeguarded through the inclusion and consultation of the health professionals during all stages of the design and development of the solution in order to develop an accurate mHealth application of quality that actually meets the needs of its users. Additionally, privacy and confidentiality issues were also taken into account since only authorized users, i.e., the nurses and doctors working in the nursing home, can have access to the information displayed in the solution. Moreover, data regarding login credentials were encrypted and the solution would only be available through an Intranet connection, i.e., a private network. However, since implementing data security protections is a difficult task to achieve, there is still some work that remains to be done in order to respond completely to the privacy requirements that are constantly emerging. In this context, it is planned to continuously improve the solution over time, through the encryption of all the data stored in the database.

## 8. Conclusions and Future Work

The project described in this manuscript aimed to introduce HICT in a Portuguese nursing home suffering from the consequences of the aging population and the usage of rudimentary methods and, subsequently, take advantage of the benefits provided by HICT in order to improve elders’ quality of life and the nursing care delivered. Therefore, considering the issues and challenges faced by the nursing home used as a case study, a mobile application was designed and developed for the health professionals working in the facility in order to help them manage the residents and assist them at the point-of-care.

In the long-term, the research team foresees that the mobile application will allow easier and faster access and manipulation of the information by the health professionals compared to the paper-based management of data, since, after some time, a paper-based process is composed of several pages. Additionally, it will help reduce time-waste and errors and, hence, improve elders’ quality of life and the nursing care delivered as well as reduce some of the work overload experienced by health professionals. Furthermore, it will enable to improve the overall performance of the nursing home and health professionals as well as optimize some of the processes occurring in the facility.

Regarding future work, it is planned to provide the necessary resources to the nursing home since, without them, the health professionals are not able to use the solution. Thus, it is intended to provide mobile devices, such as tablets and mobile phones, and a reliable wireless Internet connection, namely wireless Intranet, in order for the mobile application to be used. Afterwards, it is intended to populate the database with real data related to the health professionals and the residents. It is important to mention that the database already contains technical data (e.g., the sizes and types of urinary catheters and nasogastric tubes available and types of wounds, among others) since this information was gathered through the help of the health professionals.

On the other hand, the research team envisions designing and developing a web application to assist the mobile application and, hence, integrate some of its features. In this sense, the web application will integrate most of the features of the mobile application, allowing the health professionals to manage the residents from a computer if they prefer to do so. Additionally, it is intended to integrate into the web application a module to manage the users of the applications and another containing the clinical and performance indicators mentioned previously. However, these indicators will only be available when enough data are gathered, since, otherwise, the knowledge acquired would not be meaningful and valuable. Furthermore, it is intended to continue the expansion of the mobile application through the addition of new and relevant features. Therefore, considering the above mentioned, the research team envisions encouraging the continuous maintenance, growth, and expansion of the solution.
